# Effects of Polymorphisms in *Pepsinogen* (*PEP*), *Amylase* (*AMY*) and *Trypsin* (*TRY*) Genes on Food Habit Domestication Traits in Mandarin Fish

**DOI:** 10.3390/ijms141121504

**Published:** 2013-10-30

**Authors:** Tilin Yi, Jian Sun, Xufang Liang, Shan He, Ling Li, Zhengyong Wen, Dan Shen

**Affiliations:** Key Laboratory of Freshwater Animal Breeding, Ministry of Agriculture, College of Fisheries, Huazhong Agricultural University, Hubei Collaborative Innovation Center for Freshwater Aquaculture, Wuhan 430070, China; E-Mails: yitilin@163.com (T.Y.); sunnjjian@163.com (J.S.); laile1985@163.com (S.H.); yixingjinxian@yeah.net (L.L.); wen20084787@163.com (Z.W.); shendan@mail.hzau.edu.cn (D.S.)

**Keywords:** mandarin fish, single nucleotide polymorphisms (SNPs), *pepsinogen* (*PEP*), *amylase* (*AMY*), *trypsin* (*TRY*), food habit domestication

## Abstract

Mandarin fish (*Siniperca chuatsi*) have a peculiar feeding habit of only accepting live fish prey and refusing dead prey and artificial diets. However, previous research has shown that some individuals accept dead prey after gradual domestication. Digestive enzymes are correlated with feeding habits in fish. In the current study, SNPs in the mandarin fish genes for *pepsinogen* (*PEP*), *amylase* (*AMY*), and *trypsin* (*TRY*) were evaluated for associations with feeding habits in domesticated mandarin fish by scanning their complete genomic sequence. In total, two SNPs were found in *PEP*, one was found in *TRY*, and none were found in *AMY*. The D1(CTCC) and D5(TTTT) diplotypes in the *PEP* gene tended to show strong effects on the feeding habits of domesticated fish (*p* < 0.01). The results indicate that *PEP* may be associated with the genetic mechanism for feeding habits in mandarin fish, and the D1(CTCC) and D5(TTTT) diplotypes in the *PEP* gene may be useful markers for selecting mandarin fish with appropriate feeding habits for domestication.

## 1. Introduction

The mandarin fish (*Siniperca chuats*i), a typical carnivorous fish, is a traditionally cultured freshwater fish with high commercial value in China. However, mandarin fish have a very peculiar feeding habit. As soon as they begin to feed, they feed exclusively on live fish [1]. Because of this feeding habit, the aquaculture of mandarin fish is limited. However, Liang *et al.* [2] designed a specific training procedure for these fish and found that most mandarin fish did eventually feed on minced fish prey, although some still refused. This suggests that the feeding habits that arise during the domestication of mandarin fish may vary between individuals.

In fact, there is growing evidence that inherited differences are closely linked to feeding habits, as demonstrated in sticklebacks [3] and humans [4]. The acceptance of artificial feed is considered to be a genetic trait, such as in largemouth bass (*Micropterus salmoides Lacepede*) [5–9]. Selective breeding of mandarin fish for aquaculture should take advantage of this phenotypic difference to address challenges in feeding mandarin fish artificial diets. Single nucleotide polymorphisms (SNPs) are highly abundant markers that are typically believed to be linked to genes and impact phenotypes [10]. They represent the most frequent type of genetic variation in populations and have been widely used in gene association studies to identify alleles that potentially affect important traits in aquaculture species. After all, genetic variation is the basis of genetic adaptation to dietary environments as natural populations evolve, and the knowledge of how genes are associated with behavioral traits is increasing. Analyzing associations between genetic polymorphisms and the feeding habits of domesticated mandarin fish is an important step in understanding the genetics of complex traits. Therefore, it is of great significance to study the relationship between SNPs and the feeding habits of domesticated mandarin fish.

Digestive enzymes are important factors that influence the feeding habits of fish. Several studies have shown that the activity of digestive enzymes is correlated with the feeding habits in fish [11–16]. Moreover, digestive enzyme synthesis can be modulated by genetic factors [17,18]. *Pepsinogen* (*PEP*) is a precursor of pepsin, a gastric-specific protease that functions in digestion in the stomachs of vertebrates [19]. *Amylase* (*AMY*) is a carbohydrate hydrolytic enzyme that catalyzes the breakdown of starch into sugars. The activity of amylase differently affects a variety of feeding habits in fish [14]. *Trypsin* (*TRY*) plays a major role in protein digestion processes. It is synthesized in the cells of the pyloric caecum as the inactive precursor trypsinogen, which is secreted into the intestinal lumen and activated by enteroproteases [20]. So, we selected *PEP*, *AMY*, and *TRY* as candidate genes that may potentially influence the feeding habits of domesticated mandarin fish. The objective of this study was to identify SNPs in the *PEP*, AMY, and *TRY* genes by scanning the complete mandarin fish genomic sequence and examine the association between the observed polymorphisms and the feeding habits of domesticated mandarin fish.

## 2. Results

### 2.1. Genetic Polymorphism of *PEP*, *AMY* and *TRY* Gene

After direct sequencing by scanning the complete genomic sequence of the *PEP*, *AMY* and *TRY* genes, two SNPs (T2477C, C2528T) were found in *PEP*, one SNP (G648A) in *TRY* and no SNPs in *AMY*. SNP T2477C and SNP C2528T are located in exon 7. SNP G648A is located in exon 3. All of these SNPs are synonymous mutation.

### 2.2. Analysis of Genotype Frequencies, Allele Frequencies and Genetic Diversity Parameter at Each SNPs in *PEP* and *TRY* Gene

The results of the genotype frequencies, allele frequencies and genetic diversity parameters are given in Table 1. The major allele for SNP T2477C was T allele and for SNP C2528T was C allele in two groups. The G allele was predominant over the A allele in SNP G648A. In two groups, average expected heterozygosity (He) ranged from 0.1940 to 0.5008, polymorphism information content (PIC) was between 0.1745 and 0.3744. The Hardy-Weinberg Chi-square test showed that the two groups were in genetic equilibrium (*p* > 0.05).

### 2.3. Associations of Genotypes and Diplotypes with Food Habit Domestication Traits

Single SNP in *PEP* or *TRY* gene did not show any significant effects on food habit domestication traits in mandarin fish (data not shown). Based on the two SNPs genotyping data in *PEP* gene, five diplotypes (frequencies ≥ 3%) were observed (Table 2). Diplotype-based association analysis indicated that D1 and D5 were associated with food habit domestication traits in mandarin fish (Table 3).

## 3. Discussion

Food discrimination mechanisms of fish have been linked to their digestive tract [21] and locomotor abilities [22]. *PEP*, *AMY*, and *TRY* are important digestive enzymes. The differences between individual mandarin fish in accepting dead prey may be attributed to differences in their digestive enzymes. Hence, in this study, we selected *PEP*, *AMY*, and *TRY* as candidate genes. Single nucleotide polymorphisms (SNPs) of these three genes were examined for their effects on the feeding habits of domesticated mandarin fish. After scanning the complete genomic sequence, two SNPs were found in *PEP*, located in exon 7. One SNP, located in exon 3, was found in *TRY*, and no SNPs were found in *AMY*. Each SNP loci of *PEP* gene and *TRY* gene was not associated with the feeding habits of domesticated mandarin fish. The use of diplotypes is a more recent approach that may help elucidate the relationship between a candidate gene and a trait [23]. The association analysis showed that the D1(CTCC) and D5(TTTT) diplotypes in *PEP* were strongly associated with the feeding habits of domesticated mandarin fish (*p* < 0.01).

The activity of *AMY* in omnivorous and herbivorous species has been found to be higher than in carnivores [14]. We found no SNPs in *AMY*, and this absence may be attributable to the peculiar feeding habits of mandarin fish, which are carnivorous. *PEP* and *TRY* play important roles in protein hydrolysis. Qian [24] found that in mandarin fish, the activity of *PEP* after being activated by food is higher than the activity of *TRY* before feeding. In our study, we found that *PEP* rather than *TRY* was associated with the feeding habits of domesticated mandarin fish. These results suggest that *PEP* may play a more important role than *TRY* in the domestication of mandarin fish and that *PEP* is associated with the genetic mechanism for the feeding habits of domesticated mandarin fish.

Polymorphism information content (PIC) is a value that is commonly used in genetics as a measure of polymorphism for a marker [25]. Bostein *et al.* [26] described that a locus exhibits low polymorphism when the PIC value is less than 0.25, average polymorphism when the value is between 0.25 and 0.5, and high polymorphism when the value is higher than 0.5. Consequently, T2477C mutation in *PEP* gene showed low genetic variation, while C2528T mutation in *PEP* gene and G648A mutation in *TRY* gene exhibited average genetic variation. Higher PIC values indicate more genetic variation and more selection potential. As shown in Table 1, the PIC of nonfeeders was higher than that of feeders. This demonstrates that mandarin fish may be highly amenable to selective breeding.

## 4. Experimental Section

### 4.1. Fish and DNA Samples

The fingerlings of *Siniperca chuatsi* were obtained from Xinrong Fry Breeding Farm (Foshan, Guangdong Province, China) by artificial breeding techniques. Domestication of food habit followed the methods reported by Liang *et al* [2] using net-cages as the experimental culture in Guangdong Freshwater Fish Farm (Panyu, Guangdong Province, China). In this study, fry of India mrigal *Cirrhina mrigola* were used as the live prey fish for mandarin fish and the dead prey fish were prepared by freezing. During the training period, the fish were visually sorted into feeders and nonfeeders on the basis of plumpness or emaciation, respectively. After two weeks, we successfully got two groups: 120 feeders and 120 nonfeeders. Genomic DNA was extracted from the caudal fin ray using the TIANamp Genomic DNA Kit (Tiangen biotech, Beijing, China) according to manufacturer’s directions.

### 4.2. SNP Discovery

The full length of the *PEP*, *AMY* and *TRY* genes were directly sequenced in *Siniperca chuatsi* genomic DNA samples of 30 feeders and 30 nonfeeders using an ABI PRISM 3700 DNA analyzer (Applied Biosystems, Foster City, CA, USA). Primer sets used in the amplification and sequencing analyses were designed on the basis of the reference genome sequence for *PEP* (GenBank No. FJ797703.1), *AMY* (GenBank No. EU908272.1) and *TRY* (GenBank No. FJ373291.1). Information concerning the primers for the amplification and sequencing of the *PEP*, *AMY* and *TRY* gene is shown in Table 4. Polymerase chain reaction (PCR) conditions were optimized for each pair of primers. PCRs were performed in 25 μL reaction volumes containing 2.5 μL of 10 × PCR buffer, 1.0–3.0 mM MgCl_2_, 50 μM dNTPs, 0.4 μM of each primer, 1 U Taq polymerase (Takara Shuzo, Kyoto, Japan) and 50 ng genomic DNA. PCR conditions were as follows: initial denaturation at 94 °C for 3 min followed by 30 cycles at 94 °C for 30 s, the optimized annealing temperature (Table 4) for 30 s, 72 °C for 30 s, and then a final extension step at 72 °C for 10 min. The PCR products were purified using the TIANquick Midi Purification kit (Tiangen biotech, Beijing, China) for direct sequencing. Sequences were analyzed using DNASTAR software (version 5.0; DNASTAR Inc, Madison, WI, USA).

### 4.3. Genotyping of SNPs

All SNPs used the direct sequencing to genotype in *Siniperca chuatsi* genomic DNA samples of 120 feeders and 120 nonfeeders. The primers for PCR are shown in Table 5. The PCR protocol follows the same procedure as SNP discovery.

### 4.4. Statistical Analysis

Allelic frequencies, genotype frequencies, Hardy–Weinberg equilibrium, and observed heterozygosity (He) were statistically analyzed in the feeders and nonfeeders separately using the POPGENE software (Version 1.31; University of Alberta, Alberta, Canada). Polymorphism information content (PIC) was computed according to the following formula:

$$PIC=1-(\Sigma_{i=1}^{n}q_{i}^{2})-(\Sigma_{i=1}^{n-1}\Sigma_{j=i+1}^{n}2q_{i}^{2}q_{j}^{2})$$

*q*_i_ and *q*_j_ are the frequencies of the *i*th and *j*th alleles at one locus; *n* is the number of alleles at one locus). Associations between genotypes and diplotypes of SNPs and food habit domestication traits were performed using the chi-squared test. Results were considered to be statistically significant if bilateral *p*-values were less than 0.05. Statistical analyses were carried out using SPSS software (Version 17.0; SPSS Inc, Chicago, IL, USA).

## 5. Conclusions

In conclusion, we first identified two SNPs in *PEP*, one SNP in *TRY*, and none in *AMY* by scanning the complete genomic sequence of mandarin fish. Association analysis showed that the D1(CTCC) and D5(TTTT) diplotypes in *PEP* may be associated with the feeding habits of domesticated mandarin fish. Therefore, *PEP* may be a potential gene candidate that can affect the feeding habits of mandarin fish, and it may be useful for selectively breeding mandarin fish in the future.

## Figures and Tables

**Table 1:** Genotype frequencies and genetic diversity parameter at each Single nucleotide polymorphisms (SNPs) located in the *pepsinogen* (*PEP*) and *trypsin* (*TRY*) gene.

*PEP* T2477C									

Groups	Sample size	Genotype frequencies			Allelic frequencies		HWE	PIC	He
	
		CC	CT	TT	C	T			
Feeders	120	0.0000(0)	0.2167(26)	0.7833(94)	0.1083	0.8917	*X*^2^ = 1.6970	0.1745	0.1940
							*p* = 0.1927		
Nonfeeders	120	0.0000(0)	0.2917(35)	0.7083(85)	0.1458	0.8542	*X*^2^ = 3.3862	0.2181	0.2502
							*p* = 0.0657		

***PEP*C2528T**									

**Groups**	**Sample size**	**Genotype frequencies**			**Allelic frequencies**		**HWE**	**PIC**	**He**
	
		**CC**	**CT**	**TT**	**C**	**T**			

Feeders	120	0.6667(80)	0.2750(33)	0.0583(7)	0.8042	0.1958	*X*^2^ = 2.0823	0.2653	0.3163
							*p* = 0.1490		
Nonfeeders	120	0.5583(67)	0.3417(41)	0.1000(12)	0.7292	0.2708	*X*^2^ = 2.3333	0.3169	0.3966
							*p* = 0.1266		

***TRY*G648A**									

**Groups**	**Sample size**	**Genotype frequencies**			**Allelic frequencies**		**HWE**	**PIC**	**He**
	
		**AA**	**AG**	**GG**	**A**	**G**			

Feeders	120	0.2250(27)	0.4750(57)	0.3000(36)	0.4625	0.5375	*X*^2^ = 0.2859	0.3734	0.4993
							*p* = 0.5928		
Nonfeeders	120	0.2083(25)	0.5333(64)	0.2583(31)	0.4750	0.5250	*X*^2^ = 0.5095	0.3744	0.5008
							*p* = 0.4754		

HWE: Hardy-Weinberg equilibrium; He: gene heterozygosity; PIC: polymorphism information content.

**Table 2:** Frequencies of five diplotypes of *PEP* gene among feeders and non feeders in mandarin fish.

Diplotype	SNPs site		Frequency	
	
	T2477C	C2528T	feeders	nonfeeders
Dip1	CT	CC	0.1525	0.1709
Dip2	CT	CT	0.0508	0.1026
Dip3	TT	CC	0.5254	0.4017
Dip4	TT	CT	0.2288	0.2479
Dip5	TT	TT	0.0424	0.0769

**Table 3:** Association analysis between *PEP* diplotypes and food habit domestication traits among feeders and non-feeders in mandarin fish.

Diplotype	Feeders	Nonfeeders	*p* Value
Dip1	80	20	0.000
Non-Dip1	38	97	
Dip2	6	12	0.136
Non-Dip2	112	105	
Dip3	62	47	0.057
Non-Dip3	56	70	
Dip4	27	29	0.732
Non-Dip4	91	88	
Dip5	45	9	0.000
Non-Dip5	73	108	

**Table 4:** Primers used to amplify and sequence the genomic DNA sequence of the *Siniperca chuatsi PEP*, *AMY* and *TRY* gene.

Primer pair name	Primer sequence	Annealing Temperature (°C)	Amplified region
*PEP*-01F	TTACCAGTTAGCACCTTCAGCATG	59	5′ flanking-Intron 1
*PEP*-01R	TTGAGCCTGTACTCCTCCCATAGA		
*PEP*-02F	AAAAGGCAATGTAGCCGAACG	57	Exon 2-Exon 4
*PEP*-02R	TGTCATATCCAAGGTAGCCAGTCA		
*PEP*-03F	AGGCAAGAGCAGCACCTACAGAAA	55	Exon 4-Intron 6
*PEP*-03R	TGCAAGCCACAACCTGACCATT		
*PEP*-04F	TGGTGTTGACCCCAACCACTACTA	58	Exon 6-Exon 9
*PEP*-04R	ATAATTACAGTAGCACTG		
*AMY*-01F	GTTGCTGCTGAATCCTTG	57	5′ flanking-Intron 2
*AMY*-01R	GGTAGATGCAGGATTGTA		
*AMY*-02F	CTGTGTTGTTGCTCAGA	57	Exon 2-Exon 4
*AMY*-02R	TGAGCTGAAGCCACTAA		
*AMY*-03F	GAGTGGATGCCTGCAAG	60	Exon 4-Exon 5
*AMY*-03R	CAGTGACCCTTCCCAGATG		
*AMY*-04F	GTGCAGTTAATCTAACCCAT	58	Exon 5-Exon 6
*AMY*-04R	ATCTTGTAGAGCCTGGAAT		
*AMY*-05F	CAACCACGACAACCAGAGAG	57	Exon 6-Exon 9
*AMY*-05R	CACCTGTTTCCTTCCTTC		
*TRY*-01F	TTGTTCTGCACCACATCC	59	5′ flanking
*TRY*-01R	GTGGTGTGCCATGATGC		
*TRY*-02F	TAGAGAGTTGTCAGTCAATGC	59	5′ flanking-Exon 1
*TRY*-02R	AGGAACTTACAGGCTGCA		
*TRY*-03F	GTACGCTCAGTAGGTG	55	Exon 1-Exon 5
*TRY*-03R	CAGGAGTTGTAGTTGCAGACC		

**Table 5:** Primer sets for PCR amplification and genotyping of each SNPs located in the *PEP* and *TRY* gene.

Gene	SNPs locus		Genotyping primer
*PEP*	T2477C	forward	AGTGTTGTGACCTTCGGTGG
	C2528T	reverse	ACTCGTCTTTCTTGGCGCTT
*TRY*	G648A	forward	TAAGGTCATCCGTCACCCCA
		reverse	CATACCCCGACGTGTACCAA
